# Real-World Effectiveness of Four Types of COVID-19 Vaccines

**DOI:** 10.3390/vaccines11050985

**Published:** 2023-05-15

**Authors:** Derar H. Abdel-Qader, Hasan Abdel-Qader, Jennifer Silverthorne, Chuenjid Kongkaew, Ahmad Z. Al Meslamani, Wail Hayajneh, Adel M. Alwahadneh, Salim Hamadi, Luay Abu-Qatouseh, Riad Awad, Mohannad Al Nsour, Abdallah Alhariri, Khaldoun Shnewer, Mohammad Da’ssan, Nathir M. Obeidat, Khaldoon E. Nusair, Mothafer S. Jalamdeh, Feras Hawari, Mohammad Asad, Salah AbuRuz

**Affiliations:** 1 Faculty of Pharmacy & Medical Sciences, University of Petra, Amman 11196, Jordan; d.balawi@igec.com.au (D.H.A.-Q.); shamadi@uop.edu.jo (S.H.); labuqatouseh@uop.edu.jo (L.A.-Q.); rawad@uop.edu.jo (R.A.); 2 Al Rashid Hospital Center, Amman 11623, Jordan; 3 Ministry of Health, Amma 11118, Jordan; dbalawi@hotmail.com (H.A.-Q.); drhawari@hotmail.com (F.H.); 4 Division of Pharmacy & Optometry, The University of Manchester, Manchester M13 9PL, UK; jennifer.silverthorne@manchester.ac.uk; 5 Department of Pharmacy Practice, Naresuan University, Phitsanulok 65000, Thailand; chuenjidk@nu.ac.th; 6 AAU Health and Biomedical Research Center, Al Ain University, Abu Dhabi P.O. Box 112612, United Arab Emirates; ahmad.almeslamani@aau.ac.ae; 7 College of Pharmacy, Al Ain University, Abu Dhabi P.O. Box 64141, United Arab Emirates; 8 School of Medicine, St. Louis University, St. Louis, MO 63104, USA; wail.hayajneh@health.slu.edu; 9 School of Medicine, Jordan University of Science & Technology, Irbid 3030, Jordan; 10 Saudi Hospital, Amman 11181, Jordan; adelwahadneh031@gmail.com; 11 Eastern Mediterranean Public Health Network (EMPHNET), Amman 11195, Jordan; executive.director@emphnet.net (M.A.N.); masad@globalhealthdev.org (M.A.); 12 Fourth Generation for Medical Laboratory, Amman 11181, Jordan; aalhariri1965@gmail.com (A.A.); khaldoun@clemjo.com (K.S.); mh.dasan@smartlabsgroup.com (M.D.); 13 School of Medicine, The University of Jordan, Amman 11942, Jordan; president@ju.edu.jo; 14 King Abdullah University Hospital, Irbid 22110, Jordan; khaldon_333@yahoo.com; 15 Prince Hamza Hospital, Amman 11123, Jordan; aljalamdeh@gmail.com; 16 Department of Pharmacology and Therapeutics, College of Medicine and Health Sciences, The United Arab Emirates University, Al Ain P.O. Box 15551, United Arab Emirates; 17 Department of Clinical Pharmacy, Faculty of Pharmacy, The University of Jordan, Amman 11942, Jordan

**Keywords:** COVID-19, vaccine effectiveness, Jordan, delta variant

## Abstract

Background: There is a scarcity of evidence regarding the real-world effectiveness of coronavirus disease 2019 (COVID-19) vaccines. This was the first study to evaluate the effectiveness of four types of vaccines against asymptomatic and symptomatic infection, and COVID-19 outcomes among the general population. Methods: This was a matched comparison group quasi-experimental study conducted in Jordan between 1 January and 29 August 2021. In the first part of the study, 1200 fully vaccinated individuals were matched with 1200 unvaccinated control participants. In order to measure vaccine effectiveness, the infection rates of both vaccinated and unvaccinated groups were calculated. The second part of the study included measuring specific anti-SARS CoV-2 immune cells and antibodies. Results: BNT162b2 (Pfizer, New York, NY, USA) showed a significantly higher effectiveness against asymptomatic COVID-19 infection (91.7%) and hospitalization (99.5%) than BBIBP-CorV (Sinopharm, Beijing, China) (88.4% and 98.7%, respectively) and ChAdOx1 nCoV-19 (AstraZeneca, Cambridge, UK) (84.3%, and 98.9%, respectively). The effectiveness rates of the Sputnik V (Gamaleya Research Institute, Moscow, Russia) vaccine against asymptomatic, symptomatic, and hospitalization were 100%, 100%, and 66.7%, respectively. The highest median anti-spike (S) IgG values were seen in individuals who received BNT162b2 (2.9 AU/mL) and ChAdOx1 nCoV-19 (2.8 AU/mL) vaccines. The levels of anti-S IgG were significantly decreased after 7 months of vaccination with BNT162b2 and BBIBP-CorV. There were significant decreases in the median number of neutralizing antibodies one month and seven months after receiving BNT162b2 (from 88.5 to 75.2 4 Bioequivalent Allergen Unit per milliliter/mL), BBIBP-CorV (from 69.5 to 51.5 BAU/mL), and ChAdOx1 nCoV-19 (from 69.2 to 58.BAU/mL) vaccines. The highest percentage of T cells specific to COVID-19 vaccine was found in individuals who received BNT162b2 (88.5%). Conclusion: All four vaccines evaluated in this study showed effectiveness against asymptomatic COVID-19 infection, symptomatic infection, hospitalization, and death. Furthermore, BNT162b2, BBIBP-CorV, and ChAdOx1 nCoV-19 induced high levels of immunology markers within one month of vaccination.

## 1. Introduction

The outbreak of coronavirus disease 2019 (COVID-19) has caused millions of deaths worldwide, severely impacted the global economy, and put heavy pressure on healthcare systems [1]. One of the most important mitigating responses has been the development and manufacture of new vaccines against COVID-19. Consequently, several vaccines have been launched, with more than 13.3 billion doses administered worldwide and around three-quarters of the world’s population fully vaccinated [2]. However, those vaccines face threats posed by the continual emergence of resistant COVID-19 variants, such as Alpha (B.1.1.7), Beta (B.1.351), Delta (B.1.617.2), Gamma (P.1), and, lately, Omicron (B.1.1.529) [3,4,5].

Zheng et al. [6] conducted a meta-analysis to examine the effectiveness of COVID-19 vaccines in real-world settings in fourteen countries. Another systematic review and meta-analysis reported that mRNA vaccines were the most effective against COVID-19; however, the authors suggested considering vaccines specific to the local distribution of COVID-19 variants [7]. In this study, the effectiveness of the Moderna (mRNA-1273) vaccine was 98.1% and the effectiveness of the Pfizer-BioNTech (BNT162b2) vaccine was 91.2%. In a large retrospective cohort study, Al Kaabi et al. [8] tested the effectiveness of the Sinopharm (BBIBP-CorV) vaccine in the United Arab Emirates (UAE), and found that the effectiveness of this vaccine against mortality and hospitalization was 84.1% and 79.6%, respectively. A case-control study carried out in Japan reported the effectiveness of BNT162b2 was 79% against the Delta variant [9]. According to a large observational cohort study from the United States (US) [10], the effectiveness of mRNA-1273 against COVID-19 infection and death was 87.4% and 95.8%, respectively. Researchers also reported that vaccines were less effective in an older population [6].

BBIBP-CorV is an inactivated SARS-CoV-2 virus intended for use in special populations such as pregnant women, individuals aged 60 years old or older, and severely immune-compromised individuals because of its safety profile.

BBIBP-CorV, also known by its brand name Sinopharm, is an inactivated virus vaccine designed to stimulate an immune response without leading to disease in recipients. This vaccine uses a weak form of the SARS-CoV-2 virus in order to provide immune stimulation without risk of transmission of infection or illness in the recipients themselves. ChAdOx1 nCoV-19 (or the Oxford-AstraZeneca vaccine) is an Oxford-AstraZeneca viral-vector vaccine which employs an adenovirus as its carrier to deliver genes encoding the surface spike protein from SARS-CoV-2 virus and thus initiate an immune response in its recipient [8].

Jordan, a middle-income country in the Middle East, has actively controlled the pandemic by reinforcing public health measures, restricting movement, and encouraging public immunisation. By late 2020 and early 2021, Jordan approved the emergency use of four COVID-19 vaccines (BNT162b2, BBIBP-CorV, Sputnik V, and AstraZeneca (ChAdOx1 nCoV-19) vaccines) and started a national vaccination campaign on 13 January 2021. To receive the vaccine, all individuals had to register on the national COVID-19 vaccination platform, providing personal and medical information before receiving a vaccination appointment message on their mobile phones. A vaccination certificate was then provided following vaccination. By 17 August 2021, around 3.6 million had received the first COVID-19 vaccine dose, and 3.3 million had received the second dose. As of 1 September 2022, approximately half of the population of Jordan were fully vaccinated against COVID-19 [11].

While the results of clinical trials strongly support vaccine safety and potent vaccine efficacy, there is a scarcity of post-marketing surveillance-based studies evaluating the effectiveness of vaccines in real-world settings, which has made governmental decisions difficult. Therefore, the goal of the current study was to evaluate the effectiveness of the four approved COVID-19 vaccines in Jordan (BNT162b2, BBIBP-CorV, ChAdOx1 nCoV-19, and Sputnik V) against the Delta variant (the most predominant variant in Jordan at the time of the study) using two methodologies: first, measuring the infection rate of vaccinated individuals vs. an unvaccinated control group, and second, performing immunology tests for the levels of antibodies specific to the spike (S) and nucleocapsid (N) proteins of SARS-CoV2, neutralizing antibodies (VN), and T cells.

## 2. Method

### 2.1. Summary of Study Design

In this matched comparison group quasi-experimental study carried out in Jordan between 1 January and 29 August 2021, a group of individuals (*n* = 1200) from the fully vaccinated population were randomly chosen using cluster sampling and matched with an unvaccinated control group (*n* = 1200). After follow-up, the infection rates of both vaccinated and unvaccinated groups were calculated. In addition, the levels of different types of immune cells and antibodies were tested for the vaccinated group and, consequently, predictors for their infection rates were investigated using multivariate logistic regression. The Ethics Committee of the Jordanian Ministry of Health approved the study (REC-MOH-7722). All participants provided verbal informed consent upon phone calls and laboratory visits.

### 2.2. Study Setting, Participants and Flow

All individuals aged above 18 years old who were fully vaccinated with BNT162b2, BBIBP-CorV, ChAdOx1 nCoV-19, or Sputnik V vaccines before 17 August 2021 were eligible for inclusion. Those who had been previously infected with COVID-19 (before or during the follow-up period, confirmed by anti-N IgG) or had been diagnosed with a severe mental disorder were excluded. People with a history of autoimmune disease were also excluded. Population groups which had high internal variability in the probability of contracting infection (healthcare professionals, those who interacted with any healthcare facility during the preceding week, residents at elderly care homes, and bed-ridden or home-confined persons) were excluded.

To test the vaccine effectiveness, we used two methodological strategies. Firstly, we emulated a randomised controlled trial, in which we randomly chose fully vaccinated individuals from an active group and matched them daily with individuals who had not been vaccinated into a control group in a 1:1 ratio. To increase the power and ensure a representative sample, we aimed to enroll 1200 participants in each trial group. Vaccine effectiveness was expressed as the percentage of those who did not get infection or percentage of those who were not hospitalized compared to unvaccinated. Due to the prospective nature of the study, this trial started with a stratification of participants fully vaccinated between 1 and 10 July 2021. This stratification was conducted based on the age of participants (≤65 years versus >65 years) and vaccine type (BNT162b2, BBIBP-CorV, ChAdOx1 nCoV-19, or Sputnik V). Then, proportionate random sampling was used to recruit 1200 participants from the available strata. By 1 July 2021, 374,002 individuals received two doses of a COVID-19 vaccine, of which 192,074 (51.4%) received BNT162b2 vaccine, 121,182 (32.4%) received BBIBP-CorV, 59,540 (15.9%) received ChAdOx1 nCoV-19, and 1206 (0.3%) received Sputnik V. Consequently, we recruited 617 individuals who received BNT162b2 vaccine, 389 who received BBIBP-CorV, 191 who received ChAdOx1 nCoV-19, and 3 who received Sputnik V into the active group. To recruit controls, an expert committee designed matching criteria, namely: age (above 65 years or less than 65 years), gender (female or male), pre-existing health conditions described as risk factors for COVID-19 according to the Centers for Disease Control and Prevention, economic status (categorised into five groups), pregnancy status, place of residency (classified into inside major cities and outside major cities), history of influenza vaccination in the last season, and nationality background (classified into locals, refugees, and residents). The last criterion was established because refugees account for more than 15% of the population in Jordan. To recruit controls, data from a separate database, obtained from a medical laboratory certified by the Ministry of Health, were extracted, and individuals were phoned, checked for matching eligibility, and invited to participate in the study. We ended the follow-up for each participant after the earliest occurrence of one of the following events: the occurrence of any disease-related outcomes (symptomatic positive PCR, asymptomatic positive PCR, hospitalisation, death) or the end of the study period. The follow-up period and the laboratory testing started four weeks after receiving the second dose and lasted for six months. Data collectors consisted of fifty pharmacists and nurses.

Secondly, we assessed immune response by conducting immunology testing for anti-S IgG, VN antibodies, and COVID-19 -specific T cells for a sample of vaccinated participants who were not infected by SARS-CoV-2 in the trial, as per the methodology explained in the Supplementary Materials. Those who were included in the immunology testing were followed up with to check their COVID-19 protection.

Lastly, we ran a multivariable logistic regression model to investigate the predictors of infection of the individuals who were included in the immunological testing. In this regression, we had one dependent variable, which was the infection status (Yes/No), and many independent variables, including immunological markers. In addition, the immunology tests were repeated for vaccinated individuals after seven months of vaccination to investigate whether there was a decline in the immune cells or antibodies.

### 2.3. Data Analysis

The statistical analysis of the study data was performed using Statistical Package for the Social Sciences (SPSS) version 26. The primary statistical objective was to calculate vaccine effectiveness against infection (symptomatic and asymptomatic), hospitalization, and death after receiving two doses of any vaccine. The secondary objective was to measure the levels of specific immunologic markers (SARS-CoV-2 anti-S IgG, neutralizing antibodies, and T cells specific to COVID-19) among a sample of healthy vaccinated individuals. In this study, we calculated risk ratios for vaccination as compared with no vaccination and estimated the vaccine effectiveness as one minus the risk ratio. To measures differences in parameters of socio-demographic characteristics, Wilcoxon rank-sum and chi-square tests were used as appropriate. Covariate balance after matching was checked with the use of a plot of the mean differences between variable values (standardized for continuous variables) for the vaccinated and unvaccinated groups, with a difference of 0.1 or less considered to be acceptable. Additionally, we tested the role of covariates in the risk of infection after vaccination by running multivariate logistic regression. As different individuals received different vaccines, we used Pearl’s back-door adjustment to account for differences within the populations, conditioned on non-causal variables’ links between a predictor and an outcome. Categorical parameters were presented as absolute numbers with percentages and continuous parameters as medians with interquartile ranges. The findings of the logistic regression were listed as an adjusted odds ratio (AOR) with a 95% confidence interval (CI).

## 3. Results

The study included 1200 vaccinated participants and 1200 unvaccinated participants matched on age, gender, pregnancy status, population sector, pre-existing health conditions, place of residence, and history of influenza vaccines. Overall, more than half of the participants in vaccinated and unvaccinated groups were males (63.7% versus 63.7%, respectively), between 18 and 44 years old (57.6% versus 57.3%, respectively), and Jordanian (75.2% versus 75.5%, respectively) (Table 1). Both groups had similar distribution of baseline demographics including marital status, BMI, Charlson comorbidity score, and chronic disease. Nonetheless, vaccinated individuals smoked less, but had been previously hospitalised more than unvaccinated individuals. The number of emergency room visits prior to the study was similar across both groups.

Of the 1200 vaccinated individuals, 127 (10.6%) experienced asymptomatic COVID-19 infection, 58 (4.8%) experienced symptomatic infection, 11 (0.9%) were hospitalised, and 1 (0.08%) died within 6 months of follow-up (Table 2). Within the study population, the overall vaccines’ effectiveness rates against asymptomatic COVID-19 infection, symptomatic infection, hospitalization, and death were 89.4%, 95.2%, 99.1%, and 99.9%, respectively. Apart from Sputnik V, BNT162b2 showed a significantly higher effectiveness against asymptomatic COVID-19 infection (91.7%) and hospitalization (99.5%) than BBIBP-CorV (88.4% and 98.7%, respectively) and ChAdOx1 nCoV-19 (84.3%, and 98.9%, respectively). The effectiveness rates of Sputnik V vaccine against asymptomatic COVID-19 infection, symptomatic COVID-19 infection, and hospitalization were 100%, 100%, and 66.7%, respectively.

**Figure 1:**
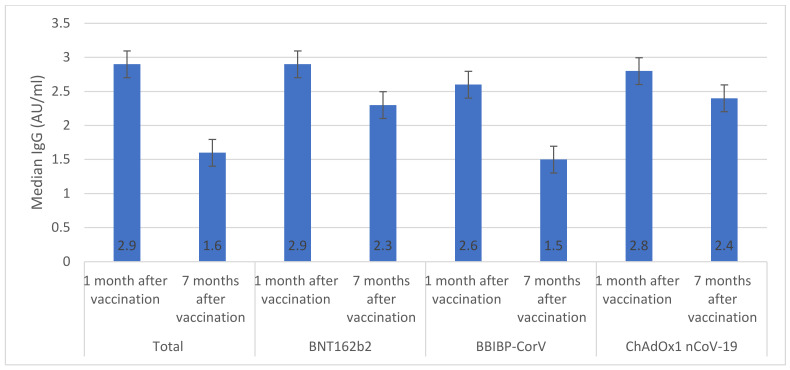
IgG response specific for the S (Spike) protein of Cov2 (AU/mL (This figure shows the levels of IgG specificity before, one month post-vaccination and seven months post-vaccination with BNT162b2, BBIP-CorV, and ChADOx nCoV-19 vaccines. Error bars represent standard deviation. If error bars of different groups don’t overlap significantly, it indicates significant variations between them in terms of IgG specificity levels).

**Figure 2:**
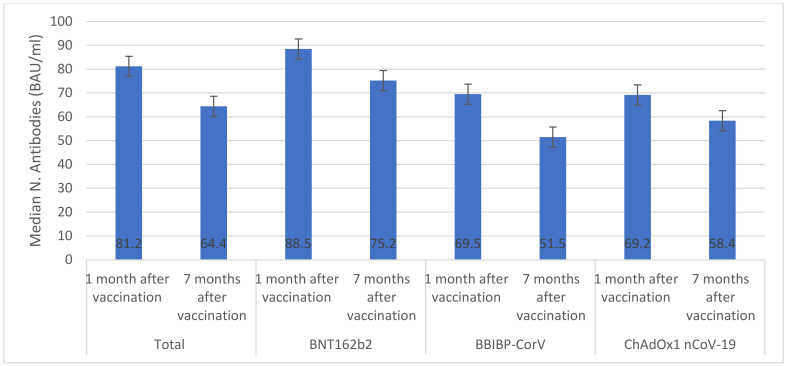
IgG response specific for the S (Spike) protein of Cov2 (BAU/mL). (This figure utilizes error bars to indicate significant differences in the median N-antibodies after administration of three vaccines).

**Figure 3:**
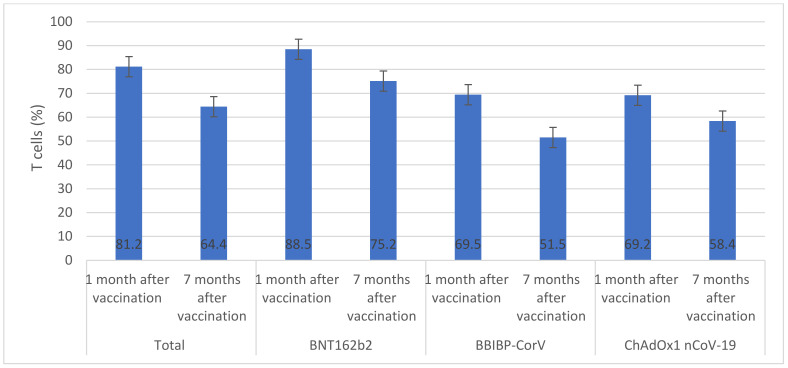
T cells across vaccines (%). (This graph presents the percentage of T cells before and after vaccination with BNT162b2, BBIP-CorV, or ChADOx nCoV-19 vaccines, using error bars to depict significant differences. The Y axis represents T cell counts while the X axis shows vaccine types. Error bars indicate significant variations among vaccine types. Non-overlapping error bars show large variance in T cell counts between them).

Overall, among vaccinated individuals who were included in the immunological assay, median anti-S IgG was significantly reduced from 2.9 AU/mL (IQR 1.9–3.1) one month after vaccination to 1.6 AU/mL (IQR 1.3–1.8) seven months after vaccination (Figure 1). The highest median anti-S IgG values were seen in individuals who received BNT162b2 (2.9 AU/mL) and ChAdOx1 nCoV-19 (2.8 AU/mL) vaccines. Only individuals who received ChAdOx1 nCoV-19 vaccine had similar anti-S IgG values one month and seven months after vaccination (*p* > 0.05). The overall median number of neutralizing antibodies significantly declined from 81.2 BAU/mL (IQR 77.5–84.6) one month after vaccination to 64.4 BAU/mL (IQR 61.3–65.8) seven months after vaccination (Figure 2). There were significant decreases in median numbers of neutralizing antibodies one month and seven months after receiving BNT162b2 (from 88.5 to 75.2 BAU/mL), BBIBP-CorV (from 69.5 to 51.5 BAU/mL), and ChAdOx1 nCoV-19 (from 69.2 to 58.4 BAU/mL) vaccines. The highest percentage of T cells specific to COVID-19 vaccines was found in individuals who received BNT162b2 (88.5%) and the lowest was found in individuals who received ChAdOx1 nCoV-19 (69.2%) (Figure 3). After seven months of vaccination, percentages of T cells specific to COVID-19 vaccines were significantly reduced in participants who were included in the immunological assay (*p* > 0.05).

We found that those with higher anti-S IgG antibody levels were more likely to avoid COVID-19 infection. Individuals who had Anti-S IgG values between 1 and 3 AU/mL had 2.1 times increased odds of, whereas those who had values over 3 AU/mL had 5.3 times more odds. Neutralizing antibody levels between 50 BAU/mL and 200 BAU/mL also provided increased odds; those who exceeded 200 BAU/mL had even greater protection (Table 3).

Other significant predictors for COVID-19 protection were T cell presence, receipt of the BNT162b2 vaccine or ChAdOx1 nCoV-19 vaccine, as well as non-diabetic status (individuals who had T cells had an increased likelihood of protection by 2.4 times); those receiving BNT162b2 had higher odds by 3.5 times and those receiving ChAdOx1 nCoV-19 had increased odds by 2.8. Additionally, non-diabetics had reduced odds by 0.6 times (meaning they were more likely to remain protected).

## 4. Discussion

This study was part of a national research project in Jordan which evaluated the safety and effectiveness of COVID-19 vaccines among the general population. The first part of the project was an active safety surveillance of four types of COVID-19 vaccines [12]. The present study provided real-world evidence of the effectiveness of four vaccines (BNT162b2, BBIBP-CorV, ChAdOx1 nCoV-19, and Sputnik V) for the prevention of COVID-19 infection, hospitalization, and death. Furthermore, it offered insights into the relationship between immunology markers (anti-S IgG, neutralizing antibodies, and T cells) and subsequent COVID-19 infection. The scientific merit of this study emerged from the diversity of vaccines included in the study and methodologies adopted for measuring vaccines’ effectiveness. While real-world investigations of the effectiveness of COVID-19 vaccines are scarce, the available studies either used one methodology for measuring the effectiveness or included one COVID-19 vaccine. Some studies included participants who had history of COVID-19 infection, which might have led to overestimation of the effectiveness of COVID-19 vaccines [8]. This study was conducted when the Delta variant was the predominant SARS-CoV-2 variant in Jordan according to the health authorities. The effectiveness calculated in this study may not include the effectiveness of the individual vaccines against SARS-CoV2 variants emerging later. Consideration should always be given to when collecting data for research studies and their subsequent interpretation, especially within an evolving pandemic such as COVID-19. With respect to Jordan at the time of study and the SARS-CoV-2 Delta variant being predominant at that point in time, calculations made during such research might not fully represent effectiveness against subsequent variants that emerged after data collection began.

As vaccines may have been designed and tested against earlier strains of SARS-CoV-2, their efficacy against new or emerging strains may differ—for instance, the Delta variant had proven more contagious and resistant to certain vaccines than earlier strains; had studies been performed prior to its appearance, then its efficacy may never have been fully evaluated.

In this study, the effectiveness of COVID-19 vaccines was different from those reported in other real-world effectiveness studies or clinical trials. The effectiveness of BBIBP-CorV against hospitalization was lower than that reported in a Phase III clinical trial [13], which reported 100% effectiveness against death and severe COVID-19 cases. This can be explained in two ways; firstly, further SARS-CoV-2 variants have appeared since clinical trials were conducted, which might have influenced the effectiveness of vaccines. Some studies have indeed reported that new variants possessed more challenging biological and epidemiological characteristics [14,15]. Secondly, this study provided real-world data of vaccines’ effectiveness, which consisted of a longer follow-up period and included participants with different demographic characteristics. Our findings were consistent, however, with a real-world study conducted in the United Arab Emirates (UAE) by Al Kaabi et al. [8] to investigate the effectiveness of BBIBP-CorV. Another study from the UAE conducted by AlHosani et al. [16] reported similar effectiveness for BBIBP-CorV against hospitalization and death.

Our findings showed greater effectiveness for BNT162b2 than other vaccines. These findings were consistent with real-world data from Scotland, which showed more than 90% effectiveness [17]. The Scottish real-world data showed 96.1% effectiveness for BNT162b2 against the Delta variant, which was the dominant variant within our study. Our study showed greater effectiveness for BNT162b2 than a study conducted in Brazil [17]. However, as the Brazilian study stated, BNT162b2 was significantly less effective against Omicron than against the Delta variant, which raised concerns about the ability of the current vaccines to offer protection against new variants.

The effectiveness of the ChAdOx1 nCoV-19 vaccine against symptomatic COVID-19 infection as reported in a Brazilian study was significantly lower (56%) than its effectiveness in our study (88%), although its effectiveness against hospitalization was much the same [18]. Whilst our comparisons are notable, it is of utmost importance to understand that a reliable comparison of the findings across different studies evaluating vaccines’ effectiveness is extremely challenging. Variations in study designs (retrospective versus prospective), follow-up period (short versus long), SARS-CoV-2 variants, methods for calculating vaccines’ effectiveness, inclusion and exclusion criteria, and sample size are examples of factors that add complexity and complicate any comparison between studies.

Several studies have reported a decrease in vaccine effectiveness over time. A US study reported that BNT162b2 effectiveness decreased from 88% after 1 month of vaccination to 47% after 5 months of vaccination [19]. Similarly, a recent study from Morocco [20], reported a significant reduction in the effectiveness of BBIBP-CorV vaccine between the first month (88%) and the sixth month (64%) after vaccination [20]. Importantly, these studies did not report immunological markers. Considerable evidence exists demonstrating how immunological markers such as neutralizing antibodies, anti-S IgG, and T cells, can provide predictability in relation to protection against SARS-CoV-2 infection. To investigate the waning in vaccines’ effectiveness, we measured anti-S IgG, neutralizing antibodies, and T cells one month and seven months after vaccination [21,22,23]. Notably, anti-S IgG levels for people given BNT162b2 and BBIBP-CorV vaccines showed a steady decrease over time, while those given ChAdOx1 nCoV-19 did not experience this decline at one month post-vaccination or at seven months post-vaccination. This could indicate that it provided extended protection against COVID-19 compared to its competitors; however, our findings indicated a significant decrease in the levels of neutralizing antibodies and T cells among vaccinated individuals who were included in the immunological assay, which may be important in retaining protection. Our study was among the first real-world investigations to follow immunology markers in vaccinated individuals. Our findings were consistent with one previous study which reported a significant decline in neutralizing antibodies in individuals who received two doses of BNT162b2 [24]. The reduction in immunology markers over time after vaccination suggests the waning effectiveness of vaccines and rationalizes the use of booster doses to prevent severe outcomes of SARS-CoV-2.

An effective immune response is critical in protecting against COVID-19 infections, and COVID-19 vaccines aim to stimulate both humoral (antibody-mediated) and cell-mediated (T cell-mediated) immunity against SARS-CoV-2 viruses. Studies have revealed that IgG antibodies and T cells induced by these vaccines correlate highly with their effectiveness at warding off infection [21].

IgG antibodies are essential in neutralizing viruses and preventing reinfection, with levels generated from COVID-19 vaccines, particularly the mRNA vaccine BNT162b2, being both high and durable, lasting several months post vaccination—suggesting a potential extended protection from exposure [25,26]. This finding gave hope for protection for individuals vaccinated against the virus following vaccination [27].

T cells play an essential part in both early and long-term responses to viral infections, from killing virus-infected cells to helping B cells produce antibodies. Recent research has indicated that SARS-CoV-2-specific T cell responses induced by COVID-19 vaccines, such as the ChAdOx1 nCoV-19 vaccine, were both high and durable; this provided evidence of protection from exposure later on as well as strong immunity in those exposed. This finding indicated the effectiveness of vaccination [27].

Overall, COVID-19 vaccines produced high levels of IgG antibodies and T cells as an indicator of their success at preventing infection. However, new variants may disrupt their efficacy over time and ongoing research must take place to monitor them against emerging variants or create vaccines to offer additional protection from emerging ones [27].

Our logistic regression findings confirmed the protective role of immunology markers against symptomatic COVID-19. This was consistent with previous studies that reported a persistent induction of T cells, anti-S IgG, and neutralizing antibodies after contracting COVID-19 infection [21,28]. Our findings also indicated that administration of BNT162b2 and ChAdOx1 nCoV-19 vaccines was associated with protection from symptomatic COVID-19. To put this in context, we found that individuals who received BNT162b2 and ChAdOx1 nCoV-19 had the highest median immunology markers, which could explain their effectiveness against symptomatic COVID-19. Our findings also indicated that having diabetes decreased the probability of being protected from COVID-19. Several studies in the literature have indicated a bidirectional relationship between COVID-19 and diabetes [29,30,31].

Our study had several limitations. Firstly, the sample size was not large compared to other studies. Nonetheless, this was the first real-world study to test the effectiveness of COVID-19 vaccines using two methods: infection rate and immunology markers. Secondly, while we maximised our efforts to identify asymptomatic and symptomatic COVID-19 infections among participants, missing data were expected given that we relied, to some extent, on self-reported information. The impact of missing data on our findings was not evaluated. Thirdly, although health officials in Jordan indicated that the Delta variant was the most prevalent SARS-CoV-2 variant at the time of the study, we could not rule out the impact of other circulating variants on our findings. Fourth, we recognise that the matching criteria adopted for this study were limited, given that smoking status, history of respiratory diseases, and social demographics (income, marital status, and educational level) were not included. Finally, no evaluation was performed of vaccines’ efficacy against COVID-19 outcomes among immunocompromised people, as their responses could differ with any vaccine administered to them.

## 5. Conclusions

BNT162b2, BBIBP-CorV, ChAdOx1 nCoV-19, and Sputnik V vaccines were effective against asymptomatic COVID-19 infection, symptomatic infection, hospitalization, and death. BNT162b2, BBIBP-CorV, and ChAdOx1 nCoV-19 induced virus-specific immune response within one month of vaccination, but the levels of these markers declined after six months of vaccination. The vaccine-induced robust immune responses may be associated with better protection against COVID-19. However, it is imperative that ongoing research and monitoring be conducted on vaccine effectiveness to assess its long-term efficacy and develop booster shots as necessary.

## Figures and Tables

**Table 1:** Baseline characteristics of the study sample.

Variable	Active Group (*n* = 1200)	Control Group (*n* = 1200)
Sex		
Female	435 (36.3%)	435 (36.3%)
Male	765 (63.7%)	765 (63.7%)
Age, years		
18–44	691 (57.6%)	687 (57.3%)
45–64	402 (33.5%)	406 (33.8%)
65–74	75 (6.3%)	70 (5.8%)
≥75	32 (2.7%)	37 (3.1%)
Population sector		
Jordanian	902 (75.2%)	906 (75.5%)
Arab	259 (21.6%)	265 (22.1%)
Non-Arab	39 (3.2%)	29 (2.4%)
Married	854 (71.2%)	836 (69.7%)
Smoker ^a^, *n* (%)	435 (36.3%)	369 (30.8%)
Obesity (BMI ≥ 30) ^b^	168 (14.0%)	172 (14.3%)
Charlson comorbidity score ^c^		
0	823 (68.6%)	819 (68.3%)
1	108 (9.0%)	107 (8.9%)
≥2	269 (22.4%)	274 (22.8%)
Chronic diseases		
Kidney disease	51 (4.3%)	53 (4.4%)
Heart disease	113 (9.7%)	116 (9.7%)
Liver disease	46 (3.8%)	42 (3.5%)
Blood disease	64 (5.3%)	65 (5.4%)
GIT disease	128 (10.7%)	124 (10.3%)
Diabetes	219 (18.3%)	215 (17.9%)
Respiratory disease	105 (8.8%)	107 (8.9%)
Bone disease	89 (7.4%)	85 (7.1%)
Number of ED visits ^c^		
0	102 (8.5%)	96 (8.0%)
1	863 (71.9%)	870 (72.5%)
≥2	235 (19.6%)	234 (19.5%)
Number of hospitalization ^c^		
0	1071 (89.3%)	968 (80.7%)
1	100 (8.3%)	187 (15.6%)
≥2	29 (2.4%)	45 (3.8%)

^a^: “an adult who has smoked 100 cigarettes in his or her lifetime and who currently smokes cigarettes”. ^b^: BMI: body mass index. ^c^: defined based on electronic medical records.

**Table 2:** Vaccine effectiveness.

	Vaccinated					Unvaccinated (*n* = 1200)
Variable	Total (*n* = 1200)	BNT162b2 (*n* = 617)	BBIBP-CorV (*n* = 389)	ChAdOx1 nCoV-19 (*n* = 191)	Sputnik V (*n* = 3)	
Asymptomatic COVID-19 infection	127	51	45	30	0	698
Symptomatic COVID-19 infection	58	25	4	11	0	482
COVID-19-related hospitalization	11	3	5	2	1	166
COVID-19-related death	1	0	0	1	0	7
Vaccine effectiveness against asymptomatic COVID-19 infection, (1-RR), %	89.4%	91.7%	88.4%	84.3%	100%	-
Vaccine effectiveness against symptomatic COVID-19 infection, (1-RR), %	95.2%	95.9%	98.9%	94.2%	100%	-
Vaccine effectiveness against hospitalization, (1-RR), %	99.1%	99.5%	98.7%	98.9%	66.7	-
Vaccine effectiveness against death, (1-RR), %	99.9%	100%	100%	99.5%	100%	-

Sputnik V. RR: risk ratio.

**Table 3:** COVID-19 protection predictors.

Independent Variables (Variables vs. Reference)	AOR	95% CI for AORs		*p* Values
		Lower	Upper	
IgG (>3 vs. less than 0.9)	5.3	2.6	5.6	0.001
IgG (1–3 vs. less than 0.9)	2.1	1.3	3.2	0.035
T cells (Presence vs. Absence)	2.4	2.1	2.5	0.002
Neutralizing antibody (20–49 vs. <20)	1.8	0.8	1.9	0.092
Neutralizing antibody (50–200 vs. <20)	2.4	1.8	3.5	0.011
Neutralizing antibody (>200 vs. <20)	4.5	3.7	5.6	0.001
Vaccine type (Pfizer vs. Sinopharm)	3.5	2.5	4.1	0.012
Vaccine type (AstraZeneca vs. Sinopharm)	2.8	1.9	3.5	0.033
Smoking (smokers vs. non-smokers)	0.7	0.6	1.6	0.201
Gender (Females vs. Males)	1.6	0.8	3.2	0.361
Diabetes (Diabetic vs. non-diabetic)	0.6	0.4	0.8	0.089
Hypertension (hypertensive vs. non-hypertensive)	0.4	0.3	2.2	0.332

## Data Availability

Data are available upon reasonable request.

## Supplementary Materials

The following supporting information can be downloaded at: https://www.mdpi.com/article/10.3390/vaccines11050985/s1.
