# Supplementary material for: Real-World Effectiveness of Four Types of COVID-19 Vaccines
Source: Vaccines (Basel). 2023 May 15;11(5):985. doi: 10.3390/vaccines11050985 (PMC10224211; doi:10.3390/vaccines11050985)
Supplement: Supplementary file 1 [file vaccines-11-00985-s001.zip › vaccines-2359715-supplementary-done.pdf]

vaccines

MDPI

Supplementary: Laboratory testing

1- SARS-COV-2 IgG S protein

enzyme-Linked Immunosorbent Assay (ELISA) is an extremely reliable method that detects antibodies directed against specific antigens, such as those present in SARS-CoV-2 viruses. Based on binding between antibodies and their specific targets (such as S proteins of these viruses) an ELISA allows researchers to use specificity between antigen and antibody interactions and the detection of specific antibodies present.

Researchers using an ELISA to detect anti-S IgG antibodies initially coat a microtiter plate with SARS-CoV-2 S protein before washing to remove unbound proteins and blocking nonspecific binding sites to minimize any unexpected interactions between substances on the plate and nonspecific binding sites on it.

Next, serum from an individual who was recently vaccinated is added to a plate and allowed to incubate; if they have produced anti-S IgG antibodies as part of vaccination response they should bind with S protein coated on plate, and after incubation washing is performed to eliminate unbound serum components from plate surface.

At this stage, a secondary antibody that specifically binds with Anti-S IgG antibodies is added, labeled using an enzyme such as horseradish peroxidase (HRP), and then washed away to ensure no unbound secondary antibody remains on the plate.

Finally, a substrate solution is added to the plate and any HRP-conjugated secondary antibodies which have bound with Anti-S IgG antibodies are catalyzed into action through chemical reaction to generate color change proportional with Anti-S IgG presence in serum sample. This color change can then be measured using a spectrophotometer or similar instrument and presented in terms of optical density (OD).

Overall, ELISA is an established and sensitive technique used to quantify Anti-S IgG antibodies present in serum samples from individuals vaccinated against SARS-CoV-2 virus. By coating microtiter plates with S protein of SARS-CoV-2 virus and using HRP conjugated secondary antibodies that detect bound Anti-S IgG antibodies bound by these secondary antibodies to identify them bound onto specific spots on these microtiter plates with HRP conjugate secondary antibodies for detection, researchers can quantify how many Anti-S IgG antibodies exist present among vaccinees as they gain insight into understanding their immune response against vaccination. Table 1 summarizes the process of laboratory testing in our study.

2. SARS-COV-2 Neutralising Antibodies.

The Chorus TRIO is an automated platform used for measuring neutralizing antibodies against SARS-CoV-2. This process utilizes a pseudovirus expressing SARS-CoV-2's spike protein as well as other markers, to test for neutralizing antibodies present. Here is how SARS-COV-2 neutralizing antibody measurements work using Chorus TRIO:

Sample Prep: Once collected, serum from an immunized individual must be separated from blood cells before being diluted to an optimal concentration and heat inactivated to eliminate any risk of viral transmission.

Addition of the Pseudovirus: Diluted serum samples are combined with pseudovirus that expresses SARS-CoV-2 spike protein for incubation at specific temperatures for a certain length of time, in order to allow neutralizing antibodies present in serum to interact with and bind with it.

After incubation, the mixture of serum and pseudovirus is added to a plate containing target cells that could potentially be infected by it; then incubated for another set period to allow infection by pseudovirus.

Measuring Infection: After incubation is complete, a plate is washed to remove any unbound pseudovirus before adding a detection reagent, which reacts with any infected target cells to produce an identifiable signal that can be measured.

Data Analysis: The Chorus TRIO software analyzes signals and calculates quantitative values representing neutralizing antibody titers present in serum samples.

Chorus TRIO's luminescence-based detection system delivers both high sensitivity and accuracy when measuring neutralizing antibodies, with validation from clinical settings as well as Emergency Use Authorization from FDA for measuring neutralizing antibodies against SARS-CoV-2.

Chorus TRIO is an automated platform which measures neutralizing antibodies to SARS-CoV-2 with pseudovirus and luminescence-based detection, in order to assess whether vaccines have produced sufficient immunity against this infectious pathogen. By measuring neutralizing antibody titers against SARS-CoV-2, researchers can gauge its success at producing an immune response and assess vaccine efficacy against it.

3- The T-SPOT.COVID test:

The T-SPOT.COVID test is an ELISPOT-based diagnostic tool that measures T cell immunity to SARS-CoV-2 infection. T cells play an essential role in protecting us against virus infections; their ability to recognize and destroy virus-infected cells

prevents its further spread and spread of infection. While antibody tests measure humoral immunity by producing antiviral antibodies against it, T-SPOT measures cell immune reactions instead.

The T-SPOT test is a highly sensitive and specific way of measuring T cell responses to SARS-CoV-2 virus infections or vaccination. It measures T cell-derived cytokines released upon encountering viral antigens; specifically targeting SARS-CoV-2 specific antigens further reduces cross-reactivity risks with other viruses or vaccines.

To conduct the T-SPOT test, blood is collected using standard phlebotomy techniques and isolated PBMCs are then isolated using standard blood collection tubes. Once processed into standard cell suspensions, certain numbers are then placed onto specially-made plates which contain specific antigens related to SARS-CoV-2; these antigens help stimulate T cell responses specific for this virus and allow accurate identification.

T cells responding to SARS-CoV-2 antigens will release cytokines that can be captured directly with antibodies coated onto plates coated with them. A secondary-labelled antibody then binds directly with these captured cytokines before being processed through detection reagents that react with them and produce spots representing where each release occurred; counting of such spots determines T cell response to SARS-CoV-2.

Grading T cell responses based on CD3 cells - which play an essential part in immune reactions - allows clinicians to easily and quickly interpret results and quickly assess severity. A no reaction response corresponds to 40%-55% CD3 cells; mild/moderate reaction (55%-665%CD3cells); while high reaction (65-89%CD3cells). This system of categorizing responses allows quick interpretation, assisting physicians determine severity quickly.

At its heart, the T-SPOT.COVID test is an accurate, sensitive, and specific diagnostic tool which measures T cell responses against SARS-CoV-2 virus infections. By measuring immune system responses using this approach, clinicians gain more information on whether vaccination has worked effectively as well as understanding effectiveness of responses from previously infected individuals or vaccinees who may already have immunity against it.

Table S1. Laboratory testing in our study

Test	SARS-COV-2 IgG S protein
Instrument	Chorus TRIO
Principle and method	In the ELISA assay, antigen is bound to the solid phase. The specific immunoglobulins are bound to antigen through incubation with diluted human serum. Subsequently, washing is performed to eliminate proteins that have not reacted; incubation is done with the conjugate, composed of antihuman IgG monoclonal antibodies conjugated to horseradish peroxidase; and peroxidase substrate is added. The blue color that develops is proportional to the concentration of specific antibodies present in the serum sample.
Normal Range	Low response: less than 1.0 AU/ml<br>Mild to moderate response: 1.0 – 1.4 AU/ml<br>High reaction: more than 1.4 AU/ml<br><br>Negative: Less than 0.9<br><br>DOUBTFUL/EQUIVOCAL: All values between 0.9 and 1.1. If the [illegible]
Sample size	50 micro liter
**Test**	**SARS-COV-2 Neutralising Antibodies**
Instrument	Chorus TRIO
Principle and method	Competitive ELISA assay (the SARS-Cov-2 anti-S1 antibodies present in the test sample compete with the tracer to occupy the binding sites of the antigen fixed on the solid phase. The unbound components are eliminated by washing, and the bound enzymatic activity is evaluated calorimetrically due to the transformation of chromogenic substrate). The intensity of the color that developed is inversely proportional to the concentration of antibodies in sample.

Normal Range	Low response: less than 140 BAU/ml<br>Mild to moderate response: 140 - 200 BAU/ml<br>High reaction: more than 200 BAU/ml<br><br>Negative : Less than 20.0 BAU/ml<br><br>Equivocal: Between 20.0 and 50.0 BAU/ml in this case repeat the test if the
Sample size	100 micro liter

The T-SPOT.COVID test:

The T-SPOT.COVID test has been developed using the standardised T-SPOT® technology testing platform to provide information on the T cell mediated immune response to SARS-CoV-2 infection15. A better understanding of the T cell response to infection is critical to providing a better understanding of the immune response to the SARS-CoV-2 virus. Measuring T cell responses to SARS-CoV-2 may be reliable at detecting infection, and thus may be an important adjunct to current diagnostic methods.

The T-SPOT test based on ELISPOT technology is normalised for both cell number and culture conditions. This means that the test standardises the number of cells and removes serum factors that could adversely affect results, making it the most sensitive and specific test for T cell measurement. A blood sample is collected using routine phlebotomy and a standard blood collection tube from which a subset of white blood cells, known as peripheral blood mononuclear cells (PBMCs), are isolated. The cells are washed, counted and normalised to create a standard cell suspension. A standard number of cells are added into specially designed plates and stimulated with antigens specific to the disease under study. Cells responding to these antigens release a chemical messenger known as a cytokine. Cytokine antibodies are used to directly capture the cytokine as it is released by the cells. A secondary labelled antibody is added and binds to the captured cytokine. A detection reagent is added and reacts with the secondary labelled antibody. This reaction produces spots, which are a footprint of where the cytokine was released. Spots are then enumerated.

T cell Response	% of CD3
No Reaction	40-55%
Mild – Moderate Response	55%-65%
High Reaction	65-90%
